# Efficacy and safety of ear acupuncture for trauma-related disorders after large-scale disasters: A protocol of systematic review

**DOI:** 10.1097/MD.0000000000016631

**Published:** 2019-08-02

**Authors:** Chan-Young Kwon, Boram Lee, Sang-Ho Kim

**Affiliations:** a Department of Clinical Korean Medicine, Graduate School, Kyung Hee University, 26, Kyungheedae-ro, Dongdaemun-gu, Seoul; b Clinical Medicine Division, Korea Institute of Oriental Medicine, 1672, Yuseong-daero, Yuseong-gu, Daejeon; c Department of Neuropsychiatry of Korean Medicine, Pohang Korean Medicine Hospital, Daegu Haany University, 411 Saecheonnyeon-daero, Nam-gu, Pohang-si, Gyeongsangbuk-do, Republic of Korea.

**Keywords:** ear acupuncture, large-scale disaster, protocol, systematic review, trauma-related disorder

## Abstract

**Background:**

This systematic review protocol describes the methods that will be used to evaluate the efficacy and safety of ear acupuncture for trauma-related disorders after large-scale disasters.

**Methods and analysis:**

The following electronic databases will be searched up to May 2019 without language or publication status restrictions: Medline, EMBASE, the Cochrane Central Register of Controlled Trials, Allied and Complementary Medicine Database, Cumulative Index to Nursing and Allied Health Literature, and PsycARTICLES. We will also search Korean, Chinese, and Japanese databases. Any clinical studies with original data related to ear acupuncture for trauma-related disorders after large-scale disaster will be included. Traumatic stress-related symptoms will be assessed as primary outcomes. Depression, anxiety, adverse events, and total effective rate will be evaluated as secondary outcomes. Two researchers will independently perform the study selection, data extraction, and assessment of study quality. Descriptive analyses of the details of participants, interventions, and outcomes for all included studies will be conducted. Data synthesis and analysis will be performed using RevMan version 5.3. The methodological quality of the included studies will be evaluated according to the study design.

**Ethics and dissemination:**

Ethical approval is not required because individual patient data are not included. The findings of this systematic review will be disseminated through a peer-reviewed publication or conference presentations.

**PROSPERO registration number:**

CRD42019134658.

## 1 Introduction

Large-scale natural disasters such as earthquakes, tsunamis, flood, and typhoons, or human-made disasters such as industrial disasters and warfare are unexpected causes that cause widespread trauma-related disorders worldwide, including post-traumatic stress disorder (PTSD). The damage caused by natural disasters is increasing every year. The number of disasters caused by natural events such as climatological, hydrological, meteorological, and geophysical events has been increased, and in 2014, the number was estimated to be nearly 1000.^[1]^ In particular, natural changes such as global warming are expected to have a greater impact on human health through disastrous events.^[2]^

According to World Mental Health Surveys, the prevalence of disaster-related PTSD, especially in high-income countries, is up to 3.8% in adults, and the risk factors include severity of exposure, history of prior stress exposure and pre-existing mental disorders.^[3]^ The prevalence can be higher in the elderly. Parker et al analyzed the occurrence of PTSD, depression, anxiety disorders, adjustment disorder, and psychological distress after natural disasters in the elderly, and found that, compared with young adults, the population was 2.11 and 1.73 times more likely to experience PTSD symptoms and adjustment disorder, respectively.^[4]^

The damage caused by disaster destroys the medical facilities, infrastructures, and human resources in the area and results in large numbers of victims, which limits the procurement of medical resources. Therefore, it is necessary to establish efficient disaster response centers and the global cooperation is also needed.^[5]^ Like in case of other trauma-related injuries, there are a lack of medical resources to manage mental health problems in disaster survivors.^[6]^ Particularly in the case of PTSD, where psychotherapy plays an important role,^[7]^ the labor-intensive nature of the intervention makes active use difficult.^[6]^ Also, the limited efficacy of pharmacotherapy used in the treatment of PTSD is also an important challenge.^[7,8]^

East Asian traditional medicines (EATMs) such as traditional Korean medicine (TKM), traditional Chinese medicine (TCM), and Kampo medicine have been widely used in Asia to solve health-related problems including mental illnesses, and the treatment modalities including herbal medicine, acupuncture, electro-acupuncture, acupressure, and massage have been successfully supplemented to large-scale disasters in Korea, China, and Japan.^[9–16]^ In addition to Asian countries, when its usage is simple, there is a possibility that acupuncture or acupressure can be effectively used in an environment where medical resources are limited such as post-disaster management. For example, in a report published in 2010, the National Acupuncture Detoxification Association (NADA) technique, a standardized auricular acupuncture protocol, was used for Kenyan refuges in Uganda.^[17]^ The interesting point in this report is that the NADA protocol is simple and easy to use, allowing participants to be educated and self-applied.^[17]^

There is a clinical basis for ear acupuncture, which may be effective for immediate pain relief, as well as psychiatric conditions such as substance abuse, insomnia, depression, and anxiety.^[18–21]^ Thus, it has been suggested that ear acupuncture may play an important role in psychiatric care as well as treating physical conditions.^[22,23]^ As in the case of Kenyan refuges, ear acupuncture, as a non-pharmacological EATM approach, may be used simply and effectively for post-disaster diseases, including trauma-related disorders. In addition, some neuroimaging studies have pointed out that stimulating auricular points can be beneficial to patients with neurological/psychiatric syndromes from a brain science standpoint.^[24,25]^

Although ear acupuncture is an easy and simple therapeutic approach that can be applied to physical and psychological problems that occur after a disaster, and such use may be valuable in such environments where medical resources are scarce (e.g., disaster), there have been no systematic reviews on the efficacy and safety of ear acupuncture for trauma-related disorders after large-scale disasters. To summarize the clinical evidence of ear acupuncture for this condition will allow healthcare practitioners to utilize this simple non-pharmacological approach based on evidence-based medicine, especially in trauma-related disorders after large-scale disaster.

## 2 Methods

### 2.1 Study registration

We have registered this protocol for systematic review in PROSPERO (registration number, CRD42019134658) on June, 2019. We will conduct the systematic review according to this protocol, but if protocol amendments occur, the dates, changes, and rationales for each amendment will be tracked in PROSPERO. We reported this protocol according to the Preferred Reporting Items for Systematic Review and Meta-Analysis Protocols 2015 statement.^[26]^

### 2.2 Data sources and search strategy

We will search the following 15 databases comprehensively from their inception dates to May, 2019: 6 English-language databases (Medline (via PubMed), EMBASE (via Elsevier), the Cochrane Central Register of Controlled Trials [CENTRAL], the Allied and Complementary Medicine Database [AMED] (via EBSCO), the Cumulative Index to Nursing and Allied Health Literature [CINAHL] (via EBSCO), and PsycARTICLES (via ProQuest)), 5 Korean-language databases (Oriental Medicine Advanced Searching Integrated System [OASIS], Koreanstudies Information Service System [KISS], Research Information Service System [RISS], Korean Medical Database [KMbase], and Korea Citation Index [KCI]), 3 Chinese-language databases (China National Knowledge Infrastructure [CNKI], Wanfang Data, and VIP), and 1 Japanese database (CiNii). In addition, we will search the reference lists of the relevant articles and perform a manual search on Google Scholar to identify additional eligible studies. In addition to journal publications, degree theses and conference proceedings will be included. There will be no restriction on language, publication date, or publication status.

The search terms will be composed to the disease term part and the intervention term part. The search strategies for the Medline are shown in Table 1 and will be modified and used similarly in the other databases.

**Table 1:**
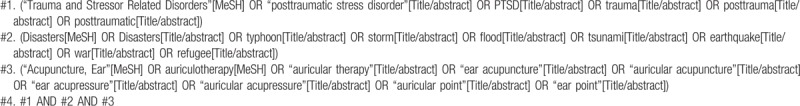
Search strategies for the Medline via PubMed.

### 2.3 Inclusion criteria

#### 2.3.1 Types of studies

Given the difficulty of a randomized controlled trial (RCT) of trauma-related disorders caused by large-scale disasters, we will include all clinical studies with original data such as case reports, retrospective studies, nonrandomized controlled trials, and RCTs. Other designs, such as in vivo, in vitro, and review articles will be excluded.

#### 2.3.2 Types of participants

We will include studies on patients with all trauma-related disorders after large-scale disaster including psychiatric disorders such as PTSD, insomnia, depressive disorders, and anxiety disorders, as well as other physical conditions such as chronic pain, diagnosed using standardized criteria such as the diagnostic and statistical manual of mental disorders (DSM), the international classification of diseases (ICD) or the Chinese classification of mental disorders (CCMD) criteria, or using clinician-administered or self-report measures. There will be no restriction on the severity of disorder, sex, age, or race of the participants. Studies will be excluded if the participants have other serious medical conditions such as cancer, liver disease, or kidney disease.

#### 2.3.3 Types of interventions

Studies using ear acupuncture for experimental intervention will be included. In this review, ear acupuncture indicates not only the application of a needle penetrating into acupuncture points, but also acupressure, which is a technique to press acupuncture points non-invasively with a finger or with a non-invasive tool such as a medicinal herb. For controlled studies, we will include studies using placebo, no treatment, and conventional medical treatments as control interventions. In addition, we will include studies involving ear acupuncture combined with other therapies as intervention of treatment group. However, controlled studies comparing different methods of ear acupuncture will be excluded because these studies cannot yield and demonstrate the net effect of ear acupuncture.

#### 2.3.4 Types of outcome measures

The primary outcome measures are traumatic stress-related symptoms measured by validated assessment tools, such as Clinician-Administered PTSD scale,^[27]^ Structured Interview for PTSD,^[28]^ or Impact of Event Scale-Revised.^[29]^

The secondary outcome measures are as follows:

(1) Depression measured by validated assessment tools such as Hamilton Rating Scale for Depression^[30]^ or Zung Self-Rating Depression Scale^[31]^
(2) Anxiety measured by validated assessment tools such as Hamilton Anxiety Rating Scale^[32]^ or Zung Self-Rating Anxiety Scale^[33]^
(3) Adverse events measured by the Treatment Emergent Symptom Scale^[34]^ or the incidence
(4) Total effective rate.

The total effective rate is a non-validated outcome measure that is processed secondarily according to certain evaluation criteria such as clinical symptom improvement or improvement rates of other quantified outcomes. In the assessment of the total effective rate, participants are generally classified as “cured” (“cured”), “markedly improved” (“markedly improved”), “improved” (“improved”), or “non-responder” (“non-responder”) after treatment. The total effective rate is calculated consistently using the following formula:

*Total effective rate = N1 + N2 + N3/N*, where *N1*, *N2*, *N3*, and *N* are the number of patients who are cured, markedly improved, improved, and who comprise the sample size, respectively.

### 2.4 Study selection

**Figure 1:**
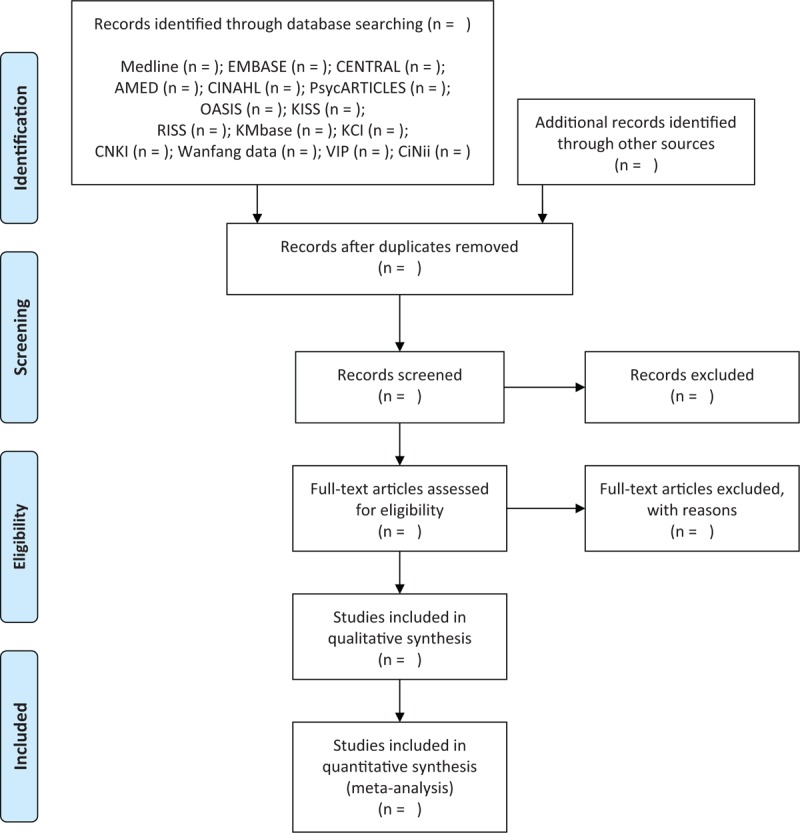
A PRISMA flow diagram of the literature screening and selection processes. AMED = Allied and Complementary Medicine Database, CENTRAL = Cochrane Central Register of Controlled Trials, CINAHL = Cumulative Index to Nursing and Allied Health Literature, CNKI = China National Knowledge Infrastructure, KCI = Korea Citation Index, KISS = Koreanstudies Information Service System, KMbase = Korean Medical Database, OASIS = Oriental Medicine Advanced Searching Integrated System, RISS = Research Information Service System.

Two researchers (CYK and BL) will independently conduct the study selection according to the above inclusion criteria. After removing duplicates, we will screen the titles and abstracts of the searched studies for first inclusion and then evaluate the full texts of the remaining eligible studies for final inclusion. Any disagreement between 2 researchers will be resolved through discussion with other researchers (SHK). Quotations from included articles will be made available to researchers using EndNote X8 (Clarivate Analytics, Philadelphia, PA), a reference management software program. Study selection process will be reported according to the Preferred Reporting Items for Systematic Reviews and Meta-Analyses statement (Fig. 1).^[35]^

### 2.5 Data extraction

Using a standardized data collection form in Excel 2016 (Microsoft, Redmond, WA), 2 researchers (CYK and BL) will independently perform and double-check the data extraction. Any discrepancy will be resolved through discussion with other researchers (SHK).

The following items will be extracted: study characteristics (author, publication year, country, and study design); approval of institutional review board; informed consent; sample size and number of dropouts; diagnostic criteria; details about the participants, intervention, and comparisons (for controlled study); duration of the intervention and follow-up; outcome measures; outcomes; and adverse events. Particularly, regarding the data on ear acupuncture such as acupuncture points and number of sessions, we will extract the data with reference to the Standards for Reporting Interventions in Clinical Trials of Acupuncture (STRICTA) recommendations.^[36]^

The extracted data will be shared among researchers using Dropbox (Dropbox, Inc., CA) folders. We will contact the corresponding authors of the included studies via e-mail to request further information if the data are insufficient or ambiguous.

### 2.6 Quality assessment

Two researchers (CYK and BL) will independently evaluate the methodological quality of the included studies. We will solve discrepancies between 2 researchers through discussion with other researchers (SHK).

For RCTs, we will use the Cochrane Collaboration's risk of bias tool to assess random sequence generation, allocation concealment, blinding of participants, personnel, and outcome assessors, completeness of outcome data, selective reporting, and other biases for each included study.^[37]^ In particular, we will assess other bias categories with particular emphasis on baseline imbalance between experimental and control groups, such as participant characteristics, including mean age, sex, or disease severity, because baseline imbalance in factors that are strongly related to outcome measures can cause bias in the estimation of the intervention effect in RCTs. We will categorize judgement relating to risk of bias into one of 3 groups: “low risk,” “unclear risk”, or “high risk.”

For nonrandomized clinical trials, we will use the Risk of Bias in Non-randomized Studies of Interventions (ROBINS-I) tool to assess the methodological quality.^[38]^

For before-after studies with no control group, and case reports/case series, we will use the tools proposed by the National Heart, Lung, and Blood Institute, namely Quality Assessment Tool for Before-After (Pre-Post) Studies with No Control Group and Quality Assessment Tool for Case Series Studies, respectively.^[39]^

Each evaluation will be recorded in an Excel 2016 (Microsoft, Redmond, WA) spreadsheet and will be shared among researchers in Dropbox (Dropbox, Inc., CA) folders.

### 2.7 Data synthesis and analysis

Descriptive analyses of the details of participants, interventions, and outcomes for all included studies will be conducted. For RCTs, meta-analysis will be performed if there are studies using the same types of intervention, comparison, and outcome measures using Review Manager version 5.3 software (Cochrane, London, UK). We will use risk ratios with 95% confidence intervals (CIs) for binary outcomes and mean difference or standardized mean difference with 95% CIs for continuous outcomes.

We will assess clinical heterogeneity by comparing the distribution of important participant factors (such as age, sex, disease severity, and morbidity period) and intervention factors (such as co-interventions and control interventions) between the included studies. Furthermore, we will assess statistical heterogeneity using both the chi-squared test and the I-squared statistic for RCTs. We will consider I-squared values ≥50% and ≥75% that are indicative of substantial and high heterogeneities, respectively. In the meta-analyses, a random effects model will be used when the heterogeneity is significant (I-squared values ≥50%), while a fixed effects model will be used when the heterogeneity is not significant. Additionally, a fixed effects model will be used when the number of studies included in the meta-analysis is lesser than five, in which case estimates of inter-study variance have poor accuracy.^[40,41]^

#### 2.7.1 Subgroup analysis

If the necessary data are available, we will conduct a subgroup analysis to explain the heterogeneity or to assess whether the treatment effects vary between subgroups according to the following criteria:

(1) the severity of trauma-related disorder; and
(2) the treatment period.

#### 2.7.2 Sensitivity analysis

If the necessary data are available, we will conduct sensitivity analyses to identify the robustness of the meta-analysis result, by excluding

(1) studies with high risks of bias and
(2) outliers that are numerically distant from the rest of the data.

#### 2.7.3 Assessment of reporting biases

If more than 10 studies are included in the meta-analysis, we will assess reporting biases, such as publication bias, using funnel plots.

### 2.8 Ethics and dissemination

Ethical approval is not required because this protocol is for a systematic review, not a clinical study. The results will be disseminated by the publication of a manuscript in a peer-reviewed journal or presentation at a relevant conference.

## 3 Discussion

Large-scale natural disasters or human-made disasters not only cause large-scale survivors with trauma-related disorders, but also limit the procurement of medical resources. Therefore, efficient response systems are urgently needed.^[5]^ Studies have shown that EATMs approaches, including auriculotherapy, may be effective in relieving symptoms of trauma-related disorders.^[9–17]^ In particular, auriculotherapy is safe and easy to use, and is known to be effective for pain, insomnia, depression, anxiety.^[18–21]^ Thus, it may be offered as an efficient approach to trauma-related disorders caused by large-scale disasters. Furthermore, there are already standardized approaches of auriculotherapy for psychiatric conditions such as the NADA protocol. However, there is no systematic and critical review to evaluate the efficacy and safety of auriculotherapy for trauma-related disorders, especially after large-scale disasters.

We believe that this systematic review will assist help healthcare practitioners, relief organizations and even survivors to utilize this simple non-pharmacological approach based on evidence-based medicine, especially in trauma-related disorders after large-scale disaster. The results will also provide useful relief strategies that could be used in future efficient disaster response centers for large-scale disasters.

## Author contributions

**Conceptualization:** Chan-Young Kwon.

**Methodology:** Chan-Young Kwon, Boram Lee.

**Supervision:** SangHo Kim.

**Writing – original draft:** Chan-Young Kwon, Boram Lee.

**Writing – review & editing:** Chan-Young Kwon, Boram Lee, SangHo Kim.

Chan-Young Kwon orcid: 0000-0003-0068-9904.
